# Primary epitheloid angiosarcoma of the pleura: an exceptional tumor location

**DOI:** 10.11604/pamj.2019.33.327.18145

**Published:** 2019-08-28

**Authors:** Mustapha Azzakhmam, Abderrahim Elktaibi, Mohamed Reda El Ochi, Mohamed Allaoui, Abderrahmane Albouzidi, Mohamed Oukabli

**Affiliations:** 1 Department of Pathology, Military Hospital of Rabat, Rabat, Morocco

**Keywords:** Angiosarcoma, epitheloid, Pleura, immunohistochemistry

## Abstract

Primary angiosarcoma of the pleura is an extremely rare tumour arising from arterial or venous pulmonary vessels of various size. It is characterized by an aggressive course and a poor prognosis. The early diagnosis is challenging due to diverse clinical and radiological manifestations. We report a case of a 70 year old male with primary right pleural epitheloid angiosarcoma. The patient had a history of a two week's progressive dyspnea. CT-scan showed a prominent thikening of the right pleura associated with pleural effusion and atelectasis. CT-scan guided by biopsy was performed and histological examination showed a tumor proliferation consisting of sheets of polygonal and epitheloid cells showing rudimentary vascular differentiation. Immunohistochemically, tumor cells were strongly positive for CD31 and Factor VIII-related antigen, negative for CD34, weakly and focally positive for EMA and Cytokeratine. The overall pathological and immunohistochemical features of the pleural specimens supported the diagnosis of epitheloid angiosarcma. The patient died after a week of discharge by pulsless ventricular tachycardia arrest. In addition, we also present a brief litterature review on pleural angiosarcoma. Our experience with this case suggests that comprehensive and sufficient sample collection and meticulous histological examination aided with immunohistochemical stains, particulary the endothelial markers, are required for accurate diagnosis of this rare malignancy.

## Introduction

Angiosarcoma is a very uncommon neoplasm derived from endothelial cells. It represents 1% to 2% of all soft tissue malignancies. The most frequent locations are skin and soft tissues. Primary pleural angiosarcoma is extremely rare; only 50 cases have been reported in the literature until 2010 [1-3]. Herein, we report a case of primary epitheloid angiosarcoma of the right pleura in a 70 years old man who had a two-weeks-history of progressive dyspnea and increase of abdominal lower limb volume. In addition, a literature review on pleural angisarcoma is included.

## Patient and observation

**Figure 1:**
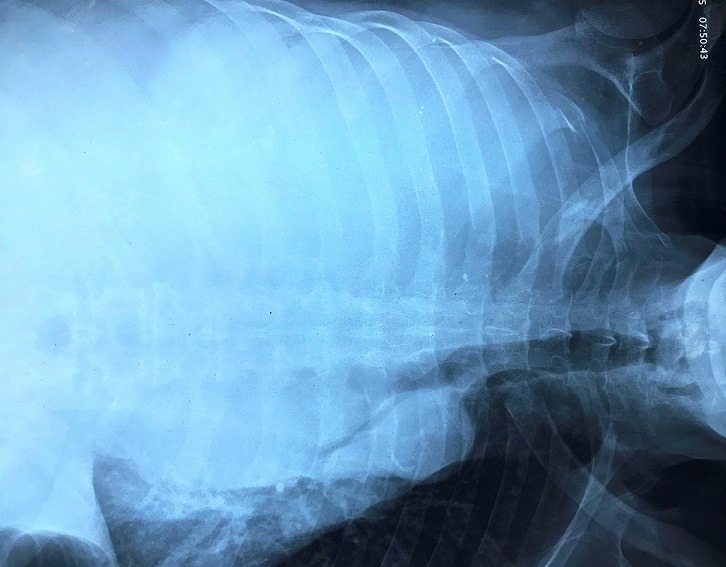
Chest radiography (X-ray) showing abundant right thorax effusion

**Figure 2:**
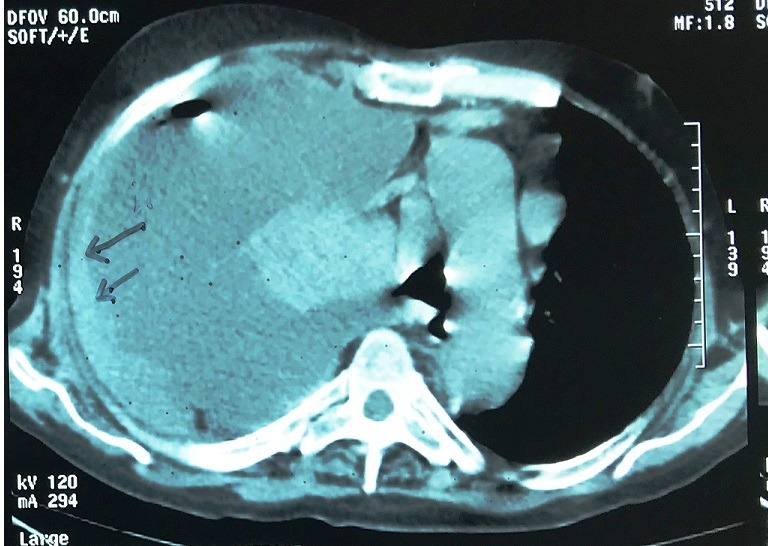
CT-scan showing a prominent thickening of the right pleura

A 70 year old man smoker was admitted to the emergency unit of the military hospital, with a history of progressive dyspnea during two weeks, and with increase of the abdominal volume and diffuse oedema of the lower limb, giving a rise to a clinical diagnosis of heart failure. He had no fever and no weight loss. His past medical history included HTA without treatment, and without diabetes or tuberculosis. On clinical examination, vesicular breath sound was diminished in right hemithorax, blood pressure was 15/10. An ECG showed sinus rhythm and left ventricle hypertrophia. An echocardiogram revealed normal function of both ventricles. Chest radiographs showed large pleural effusion (Figure 1). At right punction, approximately 3 liters of bloody fluid was drained. A CT-scan of the chest was performed and confirmed the severity of effusion, particularly in the right hemithorax, and a prominent thickening of right pleura with basilar atelectasis. Hilar and mediastinal lymphadenopathy were identified without evidence of tumor lesions in other organs (Figure 2).

**Figure 3:**
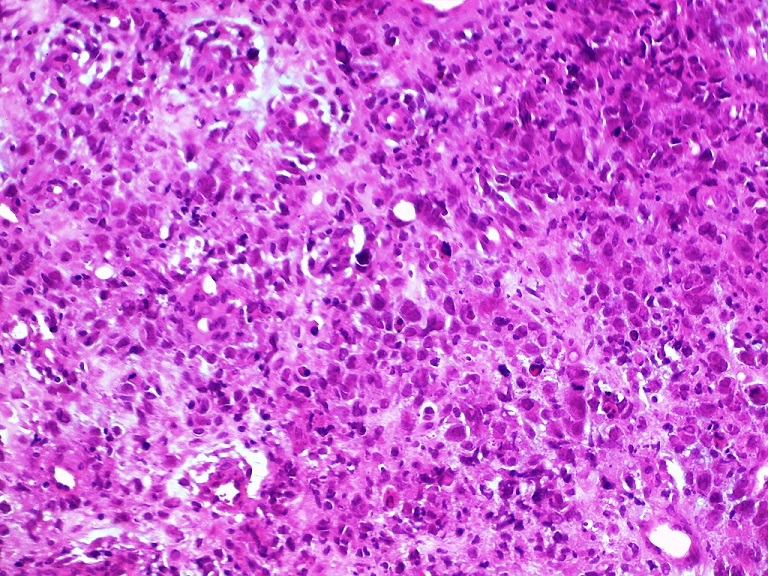
(HEX20); malignant proliferation of large epitheloid cells with marked nuclear pleomorphism and prominent nucleoli

**Figure 4:**
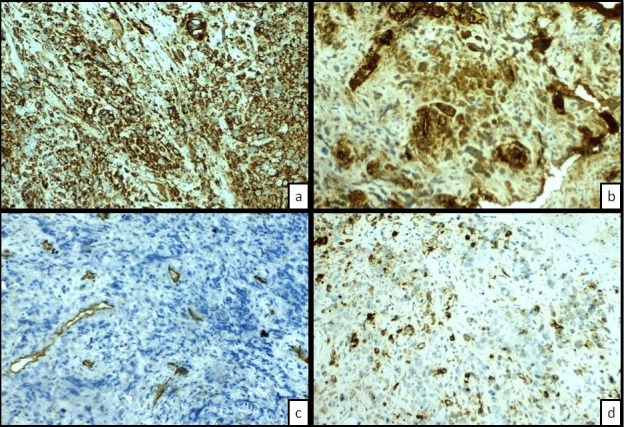
(a) diffuse strong staining with CD31; (b) diffuse strong staining with Factor VIII related antigens; (c) the negative staining of the tumoral cells with CD34; (d) weakly focal staining with EMA (magnification power x20)

**Figure 5:**
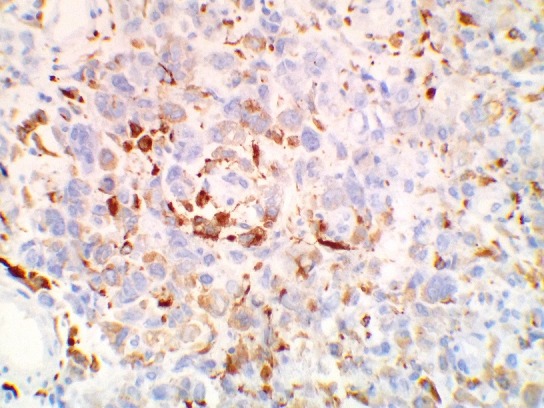
Focal cytoplasmique staining of tumoral cells with cytokeratin AE1/AE3, [HEx40]

The cytological study of pleural effusion did not reveal any definit results. A fibroscopy was performed and multiple biopsies were taken from the parietal pleura. On macroscopic examination, the received sample of pleural tissue matched with a red brown tissue of 0.4x0.3x0.2cm. The sample received was fixed with 10% buffered-formalin and stained with the hematoxilen and eosine(HE) after paraffin-embedded sections. Imunohistochemical stains have been performed on serial paraffin sections of 5u mounted on precoated slides and followed by deparrafinization using the following antibodies: TTF1, calretinin, CKAE1/AE3, Ck5/6, CD3, CD20, CD31, CD 34, FVIII-related antigens, EMA, MPO, MELAN A, HMB45, HMBE1. Histologically, it was a neoplastic proliferation consisting of large atypical epitheloid cells with abundant amphophilic cytoplasm. A marked nuclear polymorphism was observed and the nuclei showed a single prominent nucleoli and vesicular chromatine. The mitotic index was 10 per 10HPF. Intratumoral lymphocyte infiltrate was rather scanty (Figure 3). This morphological aspect was suggestive of some diagnosis, especially a pulmonary epidermoid carcinoma, a mesothelioma and even adenocarcinoma. Therefore need for immunohistochemistry was mandatory to provide an accurate diagnosis. The abovementioned antibodies were used following the diagnostic hypotesis step by step. In addition, the vasoformative pattern guided us to perform vascular markers (Table 1). Immunohistochemical analysis showed strong positivity of tumor cells for endothelial markers: CD31 and the Factor VIII-related antigens. The CD 34 was negative. The tumor cells showed focal staining for EMA and for CKAE1/AE3. They were negative for HMBE1, CK5/6, HMB45, MELAN A, calretinin, CD3, CD20, MPO, and AML (Figure 4, Figure 5). The overall pathological immunohistochemical features supported the diagnosis of a malignant epitheloid vascular tumor consistent with epitheloid.

**Table 1:** Antibodies used in the current study and results

Clone	Incubation-time	Anitbody	Stain
867G_3_/1	20 mn	TTF1	Negatif
DAK-Calret 1	20 mn	Calretinin	Negatif
Clone D5/16B4	20 mn	CK5/6	Negatif
Polyclonal F7.2.38	20 mn	CD3	Negatif
L26	20 mn	CD20	Negatif
Clone JC70A	20 mn	CD31	Intense diffuse
Clone F8/86	20 mn	F-VIII	Intense diffuse
Clone QBEnd10	20 mn	CD34	Negatif
Clone E29	20 mn	EMA	Weak Focal
Monoclonal Mouse Clone AE1/AE3	20 mn	CKAE1/AE3	Weak Focal
Polyclonal Rabbit Myelo-PO	20 mn	MPO	Negatif
A103	20 mn	Melan A	Negatif
HMB45	20 mn	HMB45	Negatif
Clone HBE1^1^	20 mn	HBE1	Negatif

## Discussion

Malignant vascular tumors correspond to rare sarcomas which can affect any part of the body [2, 4-6]. They seldom occur in serous; however, a pleural localization remains exceptional [2, 7-10]. Indeed, the pleural angiosarcomas are often secondary metastasis from primitive tumors of other sites [2, 11]. Primary pleural angiosarcoma is extremely rare; up until 2010, only 50 cases were reported in the English literature [3]. The average reported age was 55 years (in the range of 34 and 77 years), and the male/female ratio report was of 15 to 4 [1]. The clinical symptoms are not specific; pain, cough, short breathing. The most common clinical signs are represented by: chest thoracic pain, pleural pain, hemoptysy, anaemia and recurring hemothorax [3, 5, 7, 9]. The radiological signs are also not very specific, and do not allow to differentiate the pleural angiosarcoma from other pleural neoplastic-primitive, or secondary lesions. chest radiography, often shows an organic mass, a pleural thickening, and an unilateral pleural effusion [3, 5, 8, 12]. The computed tomography (CT-scan) shows a lobulated masse with defined limits, and highlighted in a heterogeneous way by the product of contrast [3, 13, 14]. The positron emission tomography (PET-Scan) have been used by some authors to evaluate the extent of the lesions [3].

The final diagnosis of the pleural angiosarcoma requires a histological and immunohistochemical study of the pleural samples. Histologically, the morphology of the pleural angiosarcoma can be spindle-shaped, which corresponds to the classical variant, or can be of epitheloide type [1, 3]. Classical angiosarcomas are characterized by vasoformative pattern consisting of irregular and anastomosed vascular channels, bordered by pleomorphic malignant endothelial cells. Conversely, this vasoformative architecture is poorly represented in the epitheloid variant. Moreover, the epitheloid angiosarcomas, are characterized by a solid sheeted nodular pattern, neoplastic epitheloid cells with abundant eosinophilic cytoplasm, and large pleomorphic nuclei, with prominent nucleoli. Mitosis, haemorrhage, and necrosis can be present in variable proportions [15, 1^6^]. The epitheloid variant accounts for 75% of the pleural angoisarcomas [3, 12]. It is often misdiagnosed as mesothelioma or adenocarcimoma [3]. Epitheloid histological variant is deemed to be an element of higher malignant potential when compared to the classical histological variant [16]. The interest of the immuno-histochemical study is crucial to distinguish the pleural angiosarcoma, from the other histologically-related neoplasms, like mesothelioma and adenocarcinoma [1, 3].

The epithelial markers (Cytokeratin, EMA), are always strongly expressed in the mesothelioma and the adenocarcinoma, whereas they are seldom expressed in the pleural angiosarcoma, and more particularly in the epitheloid variant [15]. However, both benign and malignant vascular tumors frequently express CK7 and CK14. Published studies have reported that EAGS was positive for CK8 and CK18 in approximately 50% of cases, with positivity of more than 30% of tumor cells [17]. Thus, it's important, for differential diagnosis, to consider expression of CK which is either strongly or weakly expressed in epitheloid vascular tumors but diffusely positive in carcinomas or mesotheliomas. In the current case, the expression of cytokeratin was weak and focal which is concomitant with these litterature published reports. in addition, the nagtivity of mesothelial markers expression including CK5/6, calretinin, HBME1 allow to eliminate the diagnostic hypotesis of a mesothelioma. The TTF1 was also negative allowing to eliminate an adenocarcinoma. Other suspected diagnosis been also eliminated (metastasis of melanoma, lymphoma). Although vimentine is always positive in sarcomas, whereas cytokeratins are weakly and focally expressd, a vascular tumor should be suspected when diffuse and intense vimentin positivity with negativity of mesothelioma markers (ex. calretinin), and confirmed by vascular markers. Elsewhere, the vascular markers are essential for the diagnosis of certainty of the angiosarcoma, such as CD31, CD34, and the Factor-VIII-related antigens [18].

The CD31 would be of close, the most sensitive and the most specific vascular marker, which rarely react with non-vascular tumors [2, 3, 18]. While CD34, is less specific and sensitive marker and can be expressed by other soft tissue neoplasms, in particular the solitary fibrous tumors, dermatofibrosarcoma protuberans, myofibroblastomas, epitheloid sarcomas, gastrointestinal stromal tumors, neural tumors and spindle cell lipomas [2, 17-20]. Factor VIII-related antigen often lacks immunoreactivty in vascular tumors [10] Folpe *et al.* [19], reported that the FLI-1 was expressed by 50 vascular tumours (94%) out of 53, including 20 case of angiosarcomas among 22: this study highlighted the interesting role of the FLI-1 as new nuclear marker of vascular tumors. According to the published studies, the expression of at least one endothelial marker is necessary to confirm the diagnosis of angiosarcoma. Though the CD31 is regarded as being the most specific marker and the most sensitive. In our case, the tumour showed a diffuse intense expression of both the CD31 and the factor VIII-related antigen. There was no expression of the CD34. In addition, a weak and focal expression of the epithelial antigen (EMA) and the cytokeratin; these lead to confirm the vascular-epitheloid character. Therapeutic management is based on the surgery, and radio-chemotherapy [3, 12, 18]. The vascular embolization could reduce tumor volume and reduce the bleeding, and can be realized before the surgery [19, 20]. Surgery by far is the treatment of choice for localised lesions. The radiotherapy could play a beneficial role into postoperative in the absence of diffuse lesions [12, 19, 20]. Chemotherapy has few effects, and it is generally used as palliative treatment [3]. Unfortunately, our patient died one week after evacuation of his pleural effusion, by cardiac arrest following a ventricular tachycardia, followed by asystole.

## Conclusion

In conclusion, pleural angiosarcoma is a very rare aggressive malignancy with fast fatal outcome, regardless of the various therapeutic methods. The diagnosis requires a meticulous morphological analysis, supplemented imperatively by an immunohistochemical study, including mandatory the use of vascular markers.

## Competing interests

The authors declare no competing interests.
